# Challenges and strategies for harnessing large language models in plant protection

**DOI:** 10.3389/fpls.2026.1801548

**Published:** 2026-07-07

**Authors:** Xiangshuai Li, Wenjie Shangguan, Lidong Cao, Huizhu Yuan, Xiaojing Yan

**Affiliations:** State Key Laboratory for Biology of Plant Diseases and Insect Pests, Institute of Plant Protection, Chinese Academy of Agricultural Sciences, Beijing, China

**Keywords:** artificial intelligence, domain-specific large models, integrated pest management, large language models, plant protection, smart agriculture

## Abstract

Large language models (LLMs) are increasingly being explored as intelligent tools for plant protection, with potential applications in pest and disease monitoring, control decision-making, plant quarantine, pesticide development, and precision application. However, their deployment in plant protection remains constrained by limited contextual adaptation to local agroecological conditions, insufficient reliability of single generalist models across diverse tasks, and high computational and energy costs. This Perspective summarizes current application directions of LLM-driven plant protection and analyzes the major barriers limiting their practical use. We further discuss mitigation strategies, including domain-specific fine-tuning, knowledge-graph grounding, GraphRAG, multi-agent collaboration, model compression, and hierarchical edge–cloud deployment. We argue that future LLM-based plant protection systems should be lightweight, evidence-grounded, locally adaptive, and governed by clear data-security boundaries to support sustainable and reliable agricultural decision-making.

## 1 Introduction

Scientific strategies for plant protection have significantly contributed to maintaining food security, a cornerstone of human development. However, compared to the beginning of this century, humanity now faces far more severe challenges, attributable to factors such as climate change, population growth, evolving planting practices, and ecological degradation. Moreover, the demographic shift presents a dual challenge: while demographic ageing poses a significant threat to agricultural labor stability and the sustainability of smallholder farming systems (Ren et al., 2023), the challenge in countries with younger demographic profiles lies in the need for modern technical training for the growing youth workforce to ensure food security and sustainable production (FAO, 2025). This situation compels us to critically evaluate our plant protection strategies and enhance their efficiency, a challenge that necessitates a fundamental shift toward sustainable and resilient agricultural practices (de Mûelenaere et al., 2025).

**Figure 1:**
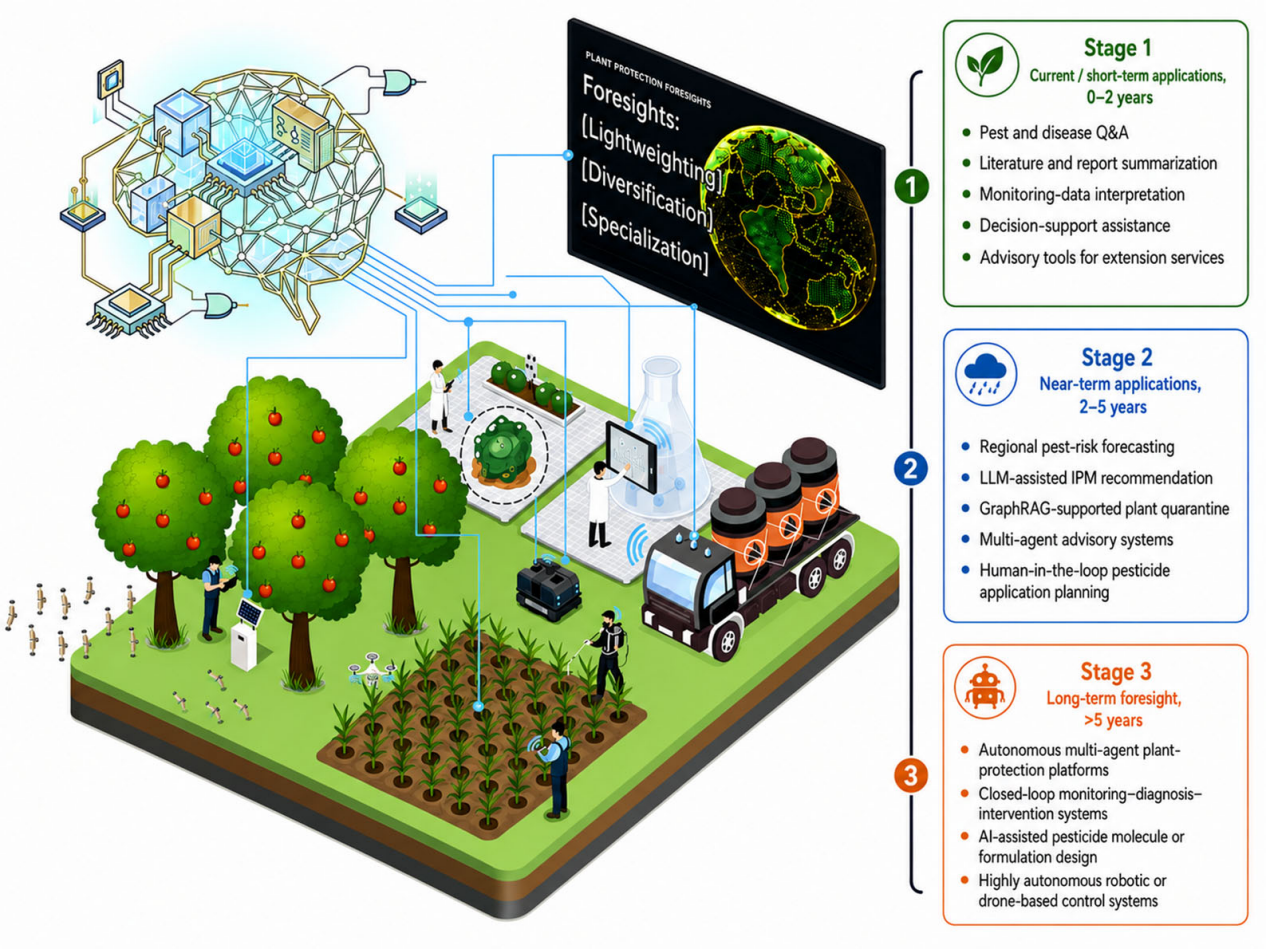
Current applications and foresights of LLMs in plant protection.

The rapid development of artificial intelligence appears to be subtly reshaping disciplinary frameworks and driving technological innovations across various fields, including agriculture and plant protection science (Lu et al., 2023). One of the most remarkable breakthroughs in artificial intelligence has been the advancement of large language models (LLMs) in machine learning. LLMs such as ChatGPT, DeepSeek, Grok, Claude, Gemini, and other comparable models, trained on vast datasets comprising billions of texts, possess the ability to emulate and generate knowledge, offering significant application potential across multiple fields. Similarly, in plant protection, LLMs hold strong appeal for agricultural scientists, entrepreneurs, agricultural technicians, and farmers, offering benefits to multiple stakeholders. Specialized models trained on extensive agricultural-related datasets are continuously emerging, providing capabilities in analysis, prediction, and strategy optimization. Nevertheless, amid this enthusiasm, we need to calmly consider whether LLMs can truly enhance plant protection? LLMs exhibit inherent limitations and cannot be seamlessly or perfectly adapted to every specialized scientific discipline (Augenstein et al., 2024). Here, we aim to highlight the key application directions for LLMs as tools in plant protection-related strategies, critically analyze current dilemmas, and propose important considerations for future developments. **Figure 1** presents an overview of how LLMs can support plant protection across major domains.

## 2 Challenges and strategies

At present, the universal characteristics of LLMs have enabled their use across various aspects of plant protection, including administrative decision-making, monitoring and early warning, scientific and technological research and development, and technology promotion (Lam et al., 2024; Li et al., 2025). Given the diversity of plant protection scenarios and the uncertainty surrounding the impact of LLMs on various strategies, we do not focus on a specific case or discuss all potential scenarios in detail. Instead, we aim to explore major technological applications driven by LLMs for smart plant protection in the context of global agricultural production challenges.

### 2.1 Pest and disease monitoring and warning

LLMs possess efficient and automated data processing capabilities. By handling both historical datasets and real-time monitoring data, including field surveys, automated collection by intelligent monitoring equipment, and pest and disease records on data platforms, they can significantly enhance efficiency and reduce manual processing costs. In modeling analysis, LLMs can integrate with other artificial intelligence algorithms to deeply analyze multi-source and multi-modal large data, enabling species identification, population dynamics prediction, and disaster level assessment. By learning from historical pest and disease trend forecasts, LLMs can combine real-time data from IoT detection equipment with model analysis results, enabling automated, intelligent forecasting across multiple scales (e.g., type, severity, and scope of impact) (Calone et al., 2025).

### 2.2 Pest and disease control

A high-precision deep learning–based pest and disease identification model collects and integrates environmental data. These data include meteorological conditions, soil properties, crop growth status, and pest and disease occurrence patterns. The integrated information is then used to develop a comprehensive model that supports crop growth assessment and pest and disease prevention and control. By establishing effective prevention indicators and thresholds, this model supports decision-making in pest and disease management. To personalize decision-making, fine-tuning the large base model can generate optimal strategies for each crop, pest, and farming season based on local conditions, thereby reducing time and economic costs while enhancing the precision and efficiency of prevention and control guidance (Zhao et al., 2024). Additionally, by integrating historical pest and disease resistance monitoring data with farmers’ feedback on prevention needs and control effectiveness, LLMs can aid in evaluating and selecting appropriate prevention and control products through data analysis and synthesis.

### 2.3 Plant quarantine

By analyzing historical data on approved crop categories and harmful organism occurrences in planting areas, LLMs can predict and flag high-risk batches, facilitating the identification of key regulated crops, quarantine organisms, and priority regulatory areas to improve the precision and efficiency of quarantine management (Abd-Elsalam and Abdel-Momen, 2024). Through automated workflows, LLMs can continuously collect and analyze global reports and scientific literature on pest occurrences in real time, enabling the monitoring of global harmful organism dynamics and the early detection of biosafety risks. Furthermore, LLM-driven risk assessment models can be developed to evaluate the introduction risk of harmful organisms by integrating key parameters such as transmission pathway weights and habitat suitability index. These models can automatically generate risk heat maps for assessing and predicting risk levels.

### 2.4 Pesticide development and application

LLMs can be utilized to develop intelligent pesticide application systems that encompass the entire process, from resistance screening and precision dispensing to formulation, adjuvant research and development, and pesticide application decision-making. The high-throughput rapid screening process improves the identification of novel pesticide active ingredients, boosts the biosynthesis efficiency of highly active compounds, and shortens the R&D cycle (Jablonka et al., 2024). During the application process, computer vision technology accurately identifies areas requiring prevention and control. Based on the severity of pests and diseases, it enables the efficient allocation of pesticide application tasks, facilitates the collaborative operation of multiple drones, and enhances the overall efficiency of pesticide application across large agricultural areas (Zhu et al., 2024a).

## 3 Challenges and mitigation strategies

**Figure 2:**
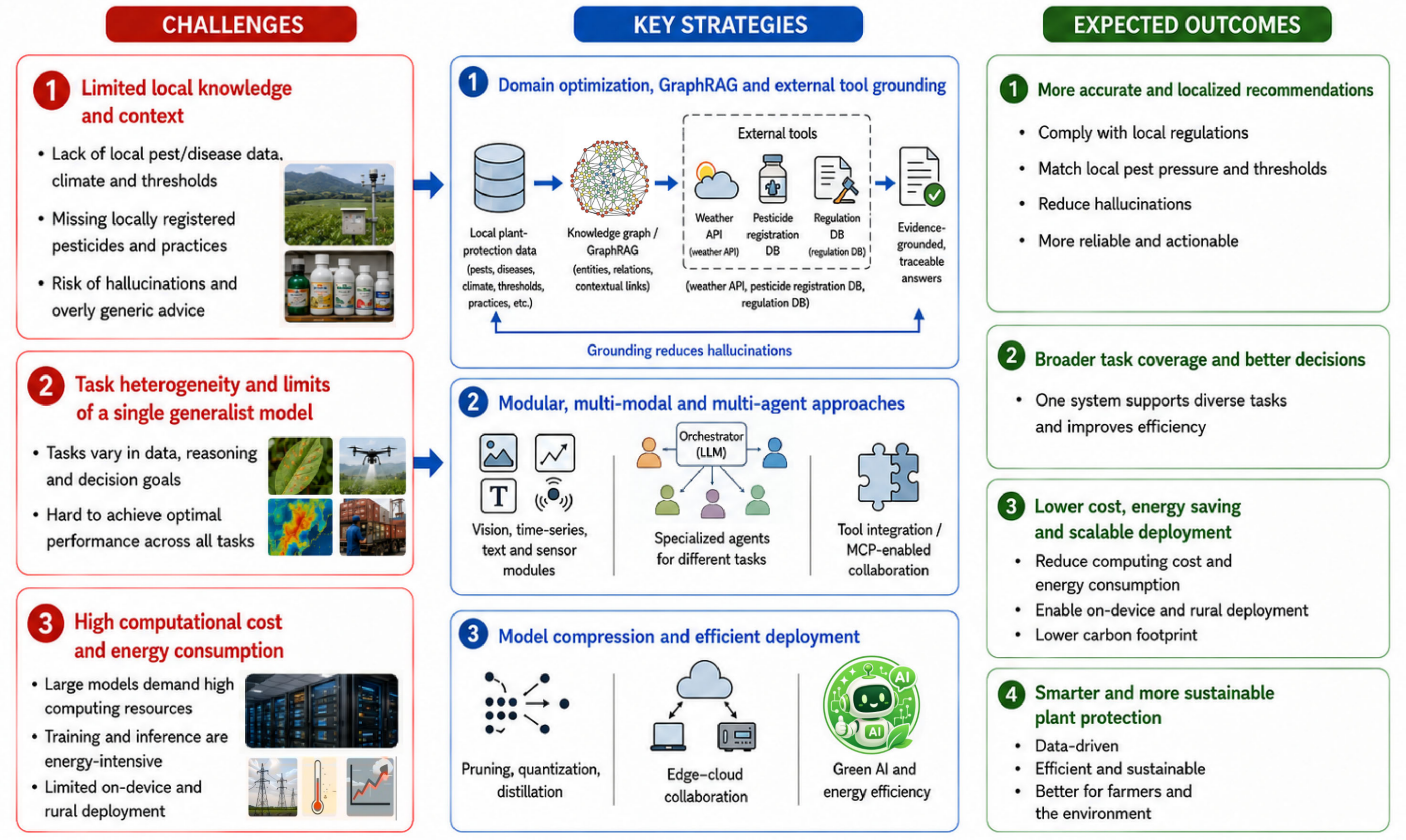
A challenge–strategy–outcome framework for LLM applications in plant protection.

Despite the significant potential exhibited by LLMs in the field of plant protection, their applications still face several challenges and limitations. Major obstacles include the insufficient adaptability of general-purpose LLMs to specific agricultural scenarios, high computational costs and energy consumption associated with these models, and the constraints imposed by relying on a single universal model to address diverse and localized issues. This section systematically discusses these challenges and explores the possibilities of overcoming these difficulties through strategies such as fine-tuning, model compression, ensemble methods, and hybrid systems. In the following subsections, we examine each challenge in detail and then describe practical strategies to mitigate it, following the “challenge–strategy–outcome” framework in **Figure 2**.

### 3.1 Insufficient local-context depth in plant protection scenarios

General-purpose LLMs are typically trained on broad but fragmented information; therefore, they may lack the contextual adaptability required for plant protection tasks. This section focuses on knowledge depth, especially regional data, local agroecological conditions, crop-specific susceptibility, registered pesticides, and management thresholds. In plant protection, expert recommendations often need to integrate localized factors such as pest and disease occurrence, pesticide formulations, application methods, cultivar susceptibility, regional action thresholds, and locally registered pesticides. Because such information is often absent from the static parameters of general-purpose LLMs, unmodified models may provide plausible but overly generic recommendations rather than locally actionable expert guidance.

Recent evaluations of ChatGPT in plant disease risk forecasting showed that GPT-4 can generate more context-aware and detailed advisory messages than GPT-3.5. However, expert assessments also indicated that both models may still produce overly generic recommendations without domain-specific adaptation involving best management practices, locally authorized pesticides, and historical treatment records (Calone et al., 2025). This limitation is consistent with recent evidence that domain-oriented instruction tuning can improve LLM performance in crop-science-related tasks (Zhang et al., 2024). Similarly, studies on deep learning–based plant leaf disease recognition have shown that models frequently exhibit limited generalization across species, environments, and symptom variability, emphasizing the necessity of domain-specific datasets and multimodal data fusion for robust real-world deployment (Zhao et al., 2025).

Recent advances in reinforcement learning–based training paradigms further suggest that the limitations of general-purpose LLMs in domain reasoning are not intrinsic but may instead reflect insufficient task-oriented optimization. Guo et al. demonstrated that DeepSeek-R1 can substantially enhance structured reasoning capabilities by explicitly incentivizing multi-step reasoning through reinforcement learning, without relying on extensive supervised chain-of-thought annotations (Guo et al., 2025). This finding suggests that, for plant protection scenarios requiring threshold-based decisions, causal inference, and risk trade-offs, reinforcement learning–driven LLMs may provide a more reliable reasoning backbone than conventionally fine-tuned models.

To address these challenges, context-specific adaptation through fine-tuning or modular augmentation becomes essential. Rather than relying on general-purpose models alone, researchers can fine-tune LLMs on curated plant protection datasets or inject domain knowledge to improve accuracy and consistency. For example, Zhang et al. introduced IPM-AgriGPT for pest and disease management, reporting improved domain performance after fine-tuning on agricultural Q&A resources (Zhang et al., 2025). Related efforts, such as the Shennong Plant Protection Multimodal LLM developed by China Agricultural University, similarly highlight the advantages of vertical-domain training for handling crop-specific terminology and region-dependent pest patterns. In parallel, modular approaches aim to reduce hallucinations by grounding generation in external knowledge. Crop GraphRAG (Wu et al., 2026). organizes pest and disease information as an agricultural knowledge graph and answers queries through entity-centric subgraph retrieval, improving factual traceability by anchoring responses to retrieved evidence. Hybrid designs that combine LLMs with knowledge graphs and symbolic reasoning have also been proposed to strengthen rigor in plant disease diagnostics (Zhao et al., 2024).

In summary, the core challenge addressed in this subsection is the depth of locally grounded knowledge rather than the breadth of plant-protection tasks. Domain-specific datasets, region-aware fine-tuning, knowledge-graph grounding, and GraphRAG can help LLMs incorporate local pest pressure, regulatory constraints, resistance patterns, and management thresholds into their responses. These approaches enable more precise, evidence-grounded, and location-aware plant-protection guidance, thereby narrowing the gap between general-purpose language generation and field-level expert decision-making.

### 3.2 Task heterogeneity and the limits of a single generalist model

A separate limitation arises from the functional heterogeneity of plant-protection tasks. In contrast to the local-context limitation discussed above, this section focuses on task breadth, including diagnosis, early warning, pesticide selection, drift prediction, resistance management, and quarantine decision support. Even when sufficient local knowledge is available, a single generalist model is unlikely to perform equally well across tasks that differ in input modalities, reasoning structures, decision objectives, and acceptable risk levels. For example, plant disease diagnosis requires visual symptom interpretation, host-pathogen knowledge, and differential diagnosis; pest forecasting requires temporal population modeling and weather-driven risk inference; pesticide application requires reasoning about dosage, formulation, nozzle selection, droplet size, drift risk, resistance management, and operator safety; and quarantine risk assessment requires pathway analysis, invasion biology, and regulatory interpretation. These tasks are not merely different local versions of the same problem. Rather, they represent distinct functional modules within plant protection. Therefore, addressing task breadth requires diversified and collaborative AI architectures composed of specialized lightweight models or agents, rather than a single universal model.

Moreover, with the advancement of integrated model systems, the Model Context Protocol (MCP) has emerged and been widely applied in multi-agent AI architectures. By offering a standardized mechanism for context sharing and coordination, this protocol effectively enhances the collaborative scheduling efficiency among multiple specialized models (Krishnan, 2025). Beyond functioning as a communication layer, MCP provides a practical bridge between static application programming interfaces and agentic plant-protection systems. Static APIs usually expose predefined functions and return fixed outputs, whereas MCP-enabled agents can dynamically discover tools, exchange task-specific context, call external databases, and coordinate multi-step reasoning processes. In plant protection, this allows an entomology agent, a pathology agent, a pesticide-science agent, and a validator agent to share structured context while maintaining task specialization.

However, such interoperability also introduces new security requirements. Farm-level data, including pesticide-use records, geolocation, crop-growth status, and pest-monitoring logs, should not be treated as unrestricted model context. MCP-based systems for plant protection should therefore define explicit security boundaries, including role-based access control, local or private-cloud deployment for sensitive data, encrypted communication between agents and servers, audit logs for tool calls, and data-minimization rules that prevent unnecessary exposure of private farm information. These safeguards are essential if MCP-based systems are to move from experimental multi-agent workflows to trusted plant-protection practice.

For instance, in crop pest management, researchers have developed an LLM-based multi-agent framework called PestMA. This system incorporates function-specific agents such as an editor, a retriever, and a validator, and optimizes the decision-making process through task decomposition and collaborative workflows. Experimental results demonstrate that this system improves the accuracy of plant protection recommendations from approximately 87% to 93%, demonstrating the significant effect of MCP in practical applications (Shi et al., 2025). This improvement may be partly attributed to hallucination suppression through retrieval, task decomposition, and validator-agent checking, rather than to language generation alone.

One promising strategy is to develop ensemble or multi-agent systems rather than relying on a single generalist model. In such systems, multiple fine-tuned LLMs or intelligent agents can be assigned to specialized domains, such as plant pathology, entomology, weed science, pesticide science, or quarantine risk assessment, and their outputs can then be integrated through routing, voting, or validation mechanisms. Evidence from other domains suggests that ensemble-based LLM frameworks can improve robustness and accuracy; for example, ensembles of medical LLMs have been reported to outperform individual models in biomedical question-answering tasks (Yang et al., 2023). Although comparable systematic benchmarks remain limited in plant protection, similar ensemble strategies could help route pest, disease, weed, and pesticide-related queries to domain-specific agents and reduce the influence of errors or biases from any single model.

A complementary strategy is to integrate LLMs with expert systems, knowledge graphs, and multi-agent workflows. Traditional rule-based Integrated Pest Management (IPM) expert systems provide structured and consistent guidance, whereas LLMs offer flexible language understanding, information synthesis, and interactive reasoning. Combining these approaches can help exploit their respective strengths while reducing individual weaknesses. For example, Zhao et al. showed that integrating LLMs with agricultural knowledge graphs can improve the efficiency and accuracy of plant disease diagnosis compared with standalone LLMs (Zhao et al., 2024). In addition, task-specific multi-agent frameworks can decompose complex plant-protection questions into retrieval, reasoning, validation, and recommendation steps. In pest management, Shi et al. reported that the PestMA multi-agent system improved the accuracy of plant-protection recommendations after validation, illustrating the potential value of collaborative agent design in this field (Shi et al., 2025). More broadly, recent reviews suggest that multi-agent LLM systems can enhance problem-solving robustness by assigning different roles to specialized agents and coordinating their outputs (Guo et al., 2024). Therefore, ensemble, hybrid, and multi-agent architectures may provide a more reliable pathway for LLM-assisted plant protection by distributing complex tasks across specialized modules and grounding recommendations in validated domain knowledge.

### 3.3 High computational costs and energy consumption

The high computational costs and energy consumption associated with LLMs pose significant challenges. Contemporary state-of-the-art LLMs typically contain billions or even hundreds of billions of parameters, demanding substantial resources for training and deployment. According to recent carbon-accounting studies, training a single large-scale language model can require substantial computational resources and result in considerable energy consumption and carbon emissions (de Vries, 2023; Luccioni et al., 2023). The training of GPT-3, which has 175 billion parameters, is estimated to have consumed approximately 1.3 million kilowatt-hours of electricity. This amount is comparable to the annual electricity usage of hundreds of average American households. Such extensive energy consumption not only incurs high financial costs but also creates environmental burdens for large-scale deployments. Moreover, the inference phase of LLMs, when deployed extensively to handle millions of queries, may consume even greater amounts of energy, requiring continuous operation of GPU or TPU clusters. De Vries observed that among widely adopted models, the energy demands during inference might even surpass those during training (de Vries, 2023). These issues pose significant obstacles to sustainable agricultural technology, as deploying massive models on edge devices such as small family-farm servers or plant protection drones is practically unrealistic. Even cloud-based deployments may become economically prohibitive for many agricultural users.

For plant-protection deployment, the feasibility of sparse activation architectures should be assessed not only by their average energy consumption but also by their operational reliability under edge conditions. Mixture-of-experts models are attractive because only a subset of expert modules is activated for each query; however, their routing mechanism, memory footprint, and communication requirements may still exceed the capacity of drone-mounted processors or low-power field devices. Therefore, a more realistic near-term strategy may be a hierarchical edge–cloud architecture: lightweight models or distilled vision-language modules can run on drones and field-station servers for rapid screening, whereas larger expert modules or MoE-based reasoning systems can be called only when high-risk cases require deeper analysis.

Consequently, there is a strong industry-wide call for the development of lighter and more efficient models tailored for practical applications. Researchers are actively exploring how to reduce model scale and computational demands without substantially compromising performance. Model compression techniques such as pruning and quantization have shown potential to transform LLMs into more compact versions, facilitating faster inference and lower energy consumption. According to a recent review by Zhu et al., reducing model weight precision can lower memory usage and computational demands by two to four times, with only minor accuracy loss (Zhu et al., 2024b). This provides a more direct quantitative basis for comparing Green AI strategies with large-scale general-purpose LLM deployment in agricultural scenarios. Similarly, knowledge distillation methods can improve inference efficiency by training a smaller “student” model to approximate the outputs or decision patterns of a larger “teacher” model. Furthermore, through domain-specific optimization, agricultural LLMs can be pruned after fine-tuning by removing redundant parameters or neurons, thereby reducing model size and inference time with limited performance loss. Additionally, some researchers advocate a “Green AI” philosophy, emphasizing energy efficiency as equally important as accuracy (Schwartz et al., 2020). Sparse activation architectures, such as mixture-of-experts models, activate only a subset of expert modules for each query and may reduce active computation compared with densely activated models.

However, efficiency-oriented compression should be evaluated against biological safety margins. In plant protection, efficiency gains should not be pursued at the expense of safety margins required for pest outbreak warning, pesticide recommendation, and resistance-risk management. For example, missed detection of a quarantine pest, early-stage disease outbreak, or pesticide-drift risk may lead to delayed intervention and serious ecological or economic consequences. Therefore, compressed, pruned, or distilled agricultural LLMs should be evaluated using task-specific metrics such as false-negative rate, recall for high-risk classes, calibration under rare-event conditions, and decision consistency under local regulatory thresholds, rather than accuracy alone.

In summary, for LLMs applied to plant protection, it is essential to pursue computationally efficient, lightweight models and appropriate deployment strategies. Such strategies should combine energy-aware model compression, hierarchical edge–cloud deployment, and risk-sensitive evaluation metrics. Doing so not only reduces usage costs but also facilitates AI-driven plant protection guidance, contributing to sustainable agricultural development.

## 4 Concluding remarks

LLMs are becoming increasingly relevant to plant protection by supporting pest and disease monitoring and warning, prevention and control decision-making, plant quarantine, and pesticide development and precision application. However, their practical deployment still depends on overcoming several challenges discussed in this Perspective. General-purpose LLMs often lack sufficient contextual adaptation for localized plant-protection decisions; single generalist models may be unreliable across heterogeneous tasks; and large-scale models impose substantial computational and energy burdens that limit sustainable deployment, especially in field and edge-device scenarios. Accordingly, future LLM-driven plant protection systems should combine domain-specific fine-tuning, knowledge-graph grounding, GraphRAG-based evidence retrieval, ensemble or multi-agent collaboration, model compression, and hierarchical edge–cloud deployment. These strategies can help develop lightweight, evidence-grounded, locally adaptive, and task-specialized LLMs for plant protection. Ultimately, the value of LLMs will depend not on their uncritical adoption, but on their careful integration with plant pathology, entomology, weed science, pesticide science, quarantine regulation, and agricultural extension. Such interdisciplinary integration will be essential for ensuring that LLM-driven plant protection improves decision-making while supporting sustainable agricultural development.

## Data Availability

Data will be made available on request.
